# Supplementary material for: Association between oral microbiome and seven types of cancers in East Asian population: a two-sample Mendelian randomization analysis
Source: Front Mol Biosci. 2023 Nov 21;10:1327893. doi: 10.3389/fmolb.2023.1327893 (PMC10702768; doi:10.3389/fmolb.2023.1327893)
Supplement: Supplementary file 1 [file DataSheet1.ZIP › Supplementary Materials/MR plots of saliva/saliva═╝/Prostate cancer/pheno.1347_to_prostate cancer_leave_one_out.pdf]

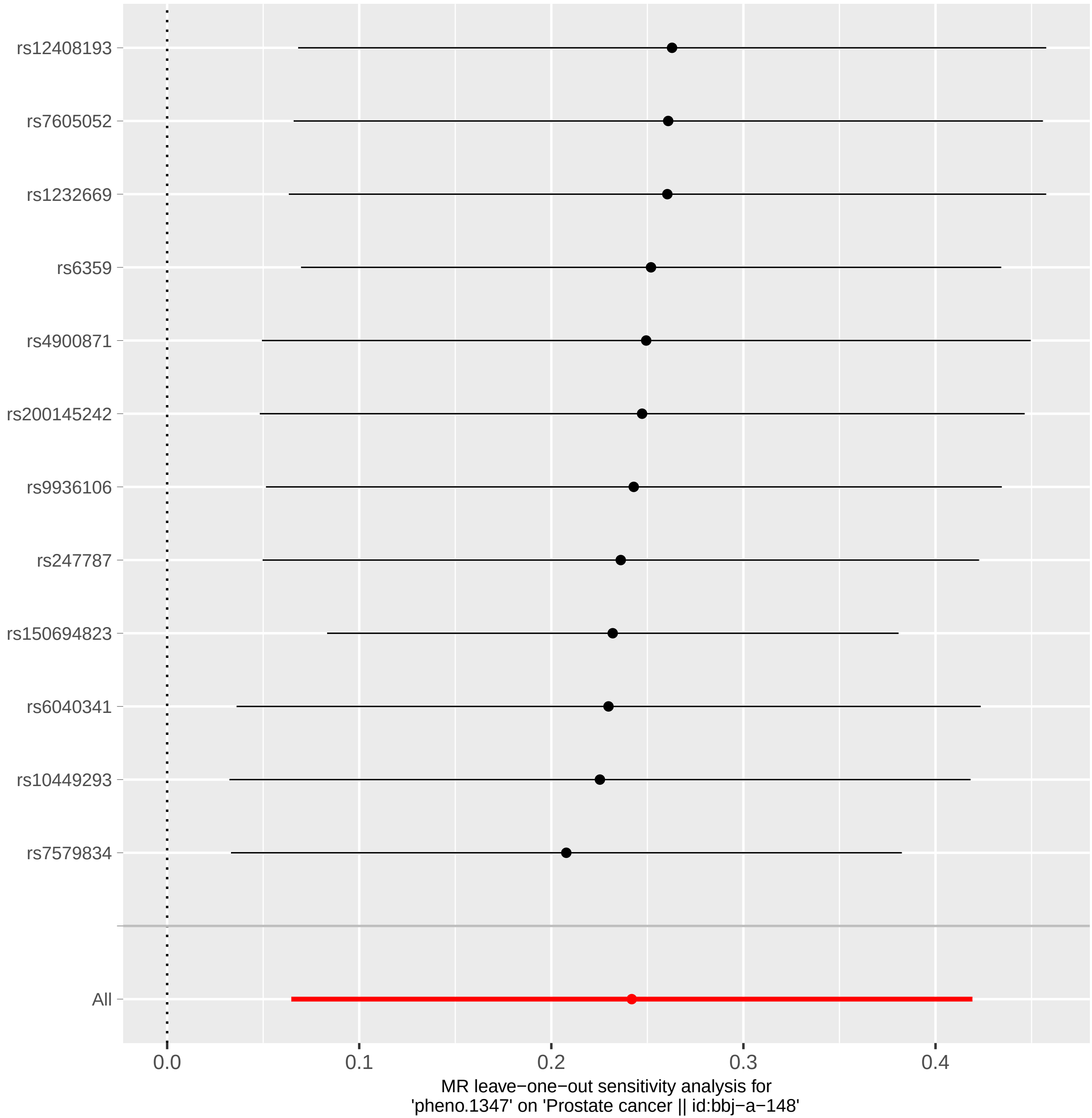
rs12408193
rs7605052
rs1232669
rs6359
rs4900871
rs200145242
rs9936106
rs247787
rs150694823
rs6040341
rs10449293
rs7579834
All
0.0
0.1
0.2
0.3
0.4
MR leave−one−out sensitivity analysis for
'pheno.1347' on 'Prostate cancer || id:bbj−a−148'
